# Novel Simulation and Analysis of Mie-Scattering Lidar for Detecting Atmospheric Turbulence Based on Non-Kolmogorov Turbulence Power Spectrum Model

**DOI:** 10.3390/e24121764

**Published:** 2022-12-01

**Authors:** Yingnan Zhang, Jiandong Mao, Juan Li, Xin Gong

**Affiliations:** 1 School of Electrical and Information Engineering, North Minzu University, North Wenchang Road, Yinchuan 750021, China; 2 Key Laboratory of Atmospheric Environment Remote Sensing of Ningxia Province, North Wenchang Road, Yinchuan 750021, China

**Keywords:** Mie-scattering lidar, non-Kolmogorov turbulence, turbulent phase screen, Gaussian beam, wave optical simulation

## Abstract

The Mie-scattering lidar can detect atmospheric turbulence intensity by using the return signals of Gaussian beams at different heights. The power spectrum method and Zernike polynomial method are used to simulate the non-Kolmogorov turbulent phase plate, respectively, and the power spectrum method with faster running speed is selected for the subsequent simulation. In order to verify the possibility of detecting atmospheric turbulence by the Mie-scattering lidar, some numerical simulations are carried out. The power spectrum method is used to simulate the propagation of the Gaussian beam from the Mie-scattering lidar in a vertical path. The propagation characteristics of the Gaussian beam using a non-Kolmogorov turbulence model are obtained by analyzing the intensity distribution and spot drift effect. The simulation results show that the scintillation index of simulation is consistent with the theoretical value trend, and the accuracy is very high, indicating that the method of atmospheric turbulence detection using Mie-scattering lidar is effective. The simulation plays a guiding role for the subsequent experimental platform construction and equipment design.

## 1. Introduction

Turbulence is a very complicated random state formed by the instability of regular laminar flow at a high velocity. Atmospheric turbulence is caused by the random motion of the atmosphere, which leads to the random fluctuation of the atmospheric refractive index. The fluctuation characteristics are mainly related to temperature gradient, humidity fluctuation and wind shear instability. When the laser propagates in atmospheric turbulence, it will cause serious laser distortion, which will affect the optical communication and other aspects [1]. Moreover, the existence of atmospheric turbulence directly affects the water–air exchange. In some arid areas with little rainfall and hot climates, atmospheric turbulence will directly affect the size of rainfall, which has a great impact on the improvement of the climate. These problems caused by atmospheric turbulence limit the development of many fields and will have very serious constraints on photoelectric systems and meteorological departments. Therefore, the detection and analysis of atmospheric turbulence is very necessary.

Theoretical analytical methods, experimental methods and numerical simulation methods are generally used to study laser propagation in turbulence. There is much research on Kolmogorov turbulence; however, there is no complete model to describe non-Kolmogorov turbulence comprehensively.

Numerical simulation methods are usually divided into the construction of turbulent phase screen and laser transmission in turbulent phase screen. In recent years, more and more studies have shown that turbulence in the upper troposphere and stratosphere of the atmosphere has deviated from Kolmogorov turbulence statistical law, that is, Kolmogorov turbulence is not the only turbulence model in the atmosphere. Beland later called this turbulence model non-Kolmogorov turbulence [2]. Therefore, in the study of laser propagation in a non-Kolmogorov turbulent atmosphere, not only the anisotropy of turbulence but also the variation of turbulence spectrum should be considered.

In recent years, many scholars have numerically simulated the non-Kolmogorov turbulent phase plate and studied its effect on lasers. In 2017, Chen et al. constructed a non-Kolmogorov turbulent phase screen using the power spectrum method based on an equivalent structure constant and discussed the propagation characteristics of Gaussian beams in it [3]. In 2018, Paulson et al. used a subharmonic phase screen based on FFT to conduct a step propagation optical simulation, demonstrating the necessity of low spatial frequency subharmonic components [4]. In 2018, Stephen et al. simulated optical propagation in non-Kolmogorov turbulence using a WOS-based stepwise phase screening method [5]. In 2020, Wang et al. proposed an IE-FBM method to generate a non-Kolmogorov turbulent phase screen, and the low-frequency and high-frequency information of the phase screen generated by this method was better when close to the theoretical value [6]. In 2021, Guan et al. derived the analytic expression of wave structure function of a plane wave and spherical wave propagation through anisotropic non-Kolmogorov turbulence in a horizontal path and simulated the influence of the internal and external scale and power ratio value of turbulence [7]. 

In addition, lidar, as an active remote sensing method, has been adopted by more researchers for the correlation measurement of atmospheric turbulence due to its advantages of strong real-time performance, high spatial and temporal resolution, and low signal pollution. In 2013, Cui et al. used light intensity scintillation lidar to detect the scintillation index and atmospheric refractive index structure constant in the horizontal direction [8]. In 2014, Tang et al. studied the detection performance of heterodyne lidar in non-Kolmogorov turbulence [9]. In 2016, Zhou et al. developed a set of range-resolved atmospheric turbulence profile lidar based on the measurement principle of atmospheric turbulence profiles using differential image shift and used it to detect atmospheric turbulence intensity profiles [10].

However, these studies generally separate theory from actual experimental detection. The numerical simulation only studies the influence of laser transmission in atmospheric turbulence theoretically and fails to obtain the relevant practical parameters of atmospheric turbulence intensity. Experimentally, there are few reports describing the use of Mie-scattering lidar for atmospheric turbulence detection. Compared with other lidar, Mie-scattering lidar is small in size, simple in operation, cost saving, easy to move, and high spatial and temporal resolution. The principle is to use the received return signal to directly detect the turbulence intensity, saving the tedious process and high cost of other image lidar processing spots, and providing a new idea for using lidar to detect the atmospheric turbulence intensity.

In order to realize the successful establishment of Mie-scattering lidar system in the next stage, this paper uses the idea of numerical simulation to simulate the system in advance. Through the simulation of the light intensity return signal, the feasibility of the system to detect atmospheric turbulence is verified and is conducive to the design of parameters of each part of the experimental equipment in the later stage.

## 2. Construction of Non-Kolmogorov Turbulent Phase Screen

The numerical simulation of light propagation in a random medium is based on the propagation equation of light and a multi-layer phase screen is used instead of a random medium. If the inhomogeneous scale of the medium is much larger than the wavelength of the light, it can be considered that there is only forward small angle scattering but no back scattering, and the propagation problem along the direction can be approximated by paraxial approximation. At this point, if the light field is expressed as $E=ue^{ikz}$, the standard parabolic equation can be obtained [11]:

(1) $$\frac{\partial u}{\partial z}=\frac{i}{2k}\nabla_{⊥}^{2}u+ikn_{1}u$$

where $\nabla_{⊥}^{2}=\frac{\partial^{2}}{\partial^{2}x}+\frac{\partial^{2}}{\partial^{2}y}$, $n_{1}$ is the refractive index fluctuation, $k=\frac{2\pi }{\lambda }$, λ is the wavelength of the light wave, which can be written in the following form:

(2) $$2ik\frac{\partial u}{\partial z}+\frac{\partial^{2}u}{\partial^{2}x}+\frac{\partial^{2}u}{\partial^{2}y}+2k^{2}n_{1}u=0$$

**Figure 1:**
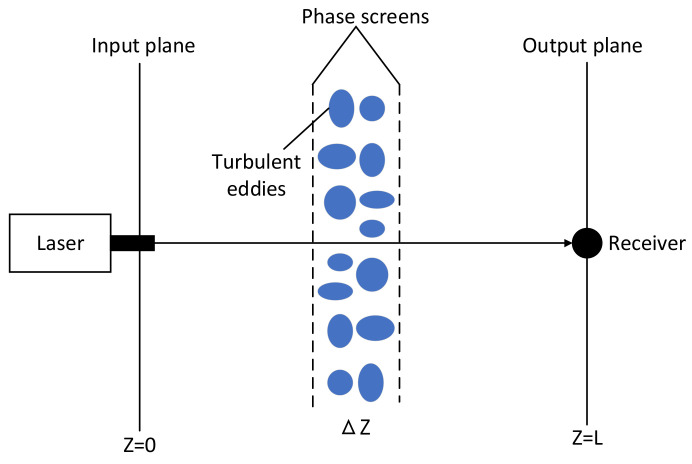
Phase screen simulation schematic diagram.

Vacuum propagation and dielectric phase modulation can be regarded as two independent and simultaneous processes when the light propagation of atmospheric turbulence is simulated by multi-layer phase screen. The continuous random medium is segmented into a series of parallel plates of thickness, through which the light field propagates and causes phase modulation, and then propagates to the next plate, as shown in Figure 1.

Then, in the propagation direction, the solution from the plane of $z=z_{i-1}$ to the plane of $z_{i}=z_{i-1}+\Delta z$ through the parallel plate of thickness Δz can be obtained by integrating the vacuum propagation with the propagation distance of Δz and phase modulation of the phase screen, and by the associated Fourier transform, we can get [1]:

(3) $$u\left(r,\;z_{i}\right)=F^{-1}\left\{\exp\left[-\frac{i\Delta z}{2k}\left(\kappa_{x}^{2}+\kappa_{y}^{2}\right)\right]F\left[e^{iS\left(r,z_{i}\right)}u\left(r,z_{i-1}\right)\right]\right\}$$

where $u\left(r,z_{i-1}\right)$ is the light field on the i−1 phase screen; $S\left(r,z_{i}\right)$ is the phase distribution on the i−1 phase screen; $\kappa_{x}$ and $\kappa_{y}$ are wave numbers in specific phase space, the units are rad/m, which are related to the scale of turbulence. $r^{2}=x^{2}+y^{2}$; F stands for Fourier transform, $F^{-1}$ stands for inverse Fourier transform. Two common methods to generate atmospheric turbulent phase screens are briefly introduced below: power spectrum method and Zernike polynomial method.

### 2.1. Power Spectrum Method

The power spectrum method is also called the Fourier Transform method (FFT) [12,13]. Its principle is to filter the power spectrum of turbulence with a complex Gaussian random number matrix, and then obtain the disturbance phase of the atmosphere by Fourier inversion. The specific process is as follows:

When the light propagates along the direction, the relationship between the two-dimensional spectrum of phase ϕ and the spatial spectral density of turbulent refractive index is [1]:

(4) $$\Phi_{\phi }\left(\kappa_{x},\kappa_{y},z\right)=2\pi k^{2}\Delta z\Phi_{n}\left(\kappa_{x},\kappa_{y},\kappa_{z}=0,z\right)$$

**Figure 2:**
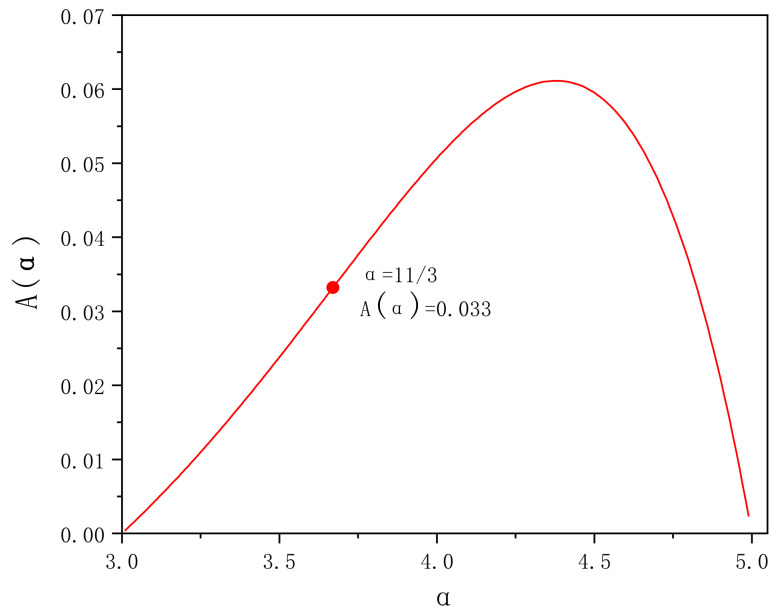
Variation of consistency function $A\left(\alpha \right)$ with power law.

For non-Kolmogorov turbulence, the three-dimensional refractive index spectrum model without considering internal and external scales is expressed as [14]:

(5) $$\Phi_{n}\left(\alpha ,\kappa \right)=A\left(\alpha \right)\tilde{C_{n}^{2}}\kappa^{-\alpha },\;\frac{1}{L_{0}}\leq \kappa \leq \frac{1}{l_{0}}$$

where $\kappa =\sqrt{x^{2}+y^{2}}$; α is the power law of three-dimensional spectrum, usually at 3<α<5 [14]. The refractive index structure constants of the atmosphere corresponding to α in a certain range are called the broad refractive index structure constants and their units are called $m^{3-\alpha }$. $A\left(\alpha \right)$ is called consistency function, whose function is to make the structure function and power spectrum of the scale index α in a certain range can be exchanged [15]. $L_{0}$ is the outer scale of turbulence and $l_{0}$ is the inner scale of turbulence.

(6) $$A\left(\alpha \right)=\frac{\Gamma \left(\alpha -1\right)}{4\pi^{2}}\cos\left(\frac{\alpha \pi }{2}\right),3<\alpha <5$$

where the $\Gamma \left(\;\right)$ is the gamma function. The consistency function $A\left(\alpha \right)$ is shown in Figure 2.

When α=11/3, $A\left(\alpha \right)=0.033$, $\tilde{C_{n}^{2}}=C_{n}^{2}$, and the unit is $m^{-2/3}$. Then, the non-Kolmogorov turbulence spectrum is transformed into the Kolmogorov turbulence spectrum [11]:

(7) $$\Phi_{n}\left(\kappa \right)=0.033C_{n}^{2}\kappa^{-\frac{11}{3}},\frac{1}{L_{0}}\leq \kappa \leq \frac{1}{l_{0}}$$

Based on the two-dimensional spectrum of phase ϕ, we can construct a two-dimensional complex random field in the phase space through the complex Gaussian random number matrix filtering:

(8) $$\tilde{\Phi }\left(\kappa_{x},\kappa_{y}\right)=a_{R}\sqrt{\Phi_{\phi }\left(\kappa_{x},\kappa_{y},z\right)}$$

(9) $$a_{R}=A_{R}+iB_{R}$$

where $a_{R}$ is a complex Gaussian random number matrix, $A_{R}$ and $B_{R}$ are random numbers with mean value of 0 and variance of 1 for both real and imaginary parts, respectively. A two-dimensional random phase field can be obtained by Fourier transform of the two-dimensional complex random field in the phase space, after discretization, we can get:

(10) $$\tilde{\Phi }\left(p\Delta \kappa ,q\Delta \kappa \right)=\frac{a_{R}\sqrt{\Phi_{\phi }\left(p\Delta \kappa ,q\Delta \kappa \right)}}{\Delta \kappa }$$

(11) $$\Delta \kappa =\frac{2\pi }{N\Delta x}$$

where N is the number of grids in the direction of length and width of phase screen, here, the length and width of the phase screen are set to be equal, then the phase screen is divided into N×N square grids; Δx is the width of each grid; Δκ is the wave number interval of the corresponding phase space; p and q are integers.

The phase distribution in real space can be obtained by FFT of Equation (10):

(12) $$\Phi \left(p\Delta x,q\Delta x\right)=\frac{1}{N^{2}}\sum_{m=0}^{N-1}\sum_{n=0}^{N-1}\tilde{\Phi }\left(m\Delta \kappa ,n\Delta \kappa \right)\exp\left[-\frac{2\pi i\left(mp+nq\right)}{N}\right]$$

Although the power spectrum method is simple to simulate turbulent phase screen, the generated phase screen has the disadvantage of insufficient low-frequency information. The minimum spatial frequency of the phase screen generated by the power spectrum method is $f_{min}=\Delta f=1/L$, L is the width of the phase screen, and the maximum spatial frequency is $f_{max}=N\Delta f/2=1/\left(2\Delta x\right)$, which does not include the power spectrum corresponding to low frequency components of $\left(0,\Delta f_{x}\right)$ and $\left(0,\Delta f_{y}\right)$.$\Delta f_{x}$, $\Delta f_{y}$ are the x and y directions of the phase screen, respectively, namely the long and wide directions of the phase screen. 

According to this situation, Lane et al. proposed a method of stacking low-frequency sub-harmonics to compensate the generated phase screen [16]. The principle is to divide the high frequency part of the power spectrum into nine small square regions with equal area, and the sampling points are distributed in the remaining eight small regions except the central point to form a sub-harmonic grid. In this way, the original high-frequency sampling part is replaced by the sub-harmonic grid, and the p-order harmonic generated by this method is expressed as:

(13) $$\Phi_{sh}\left(p^{\prime }\Delta x,q^{\prime }\Delta x\right)=\sum_{p=1}^{N_{p}}\sum_{m^{\prime }=0}^{1}\sum_{n^{\prime }=0}^{1}\tilde{\Phi }\left(p^{\prime }\Delta x,q^{\prime }\Delta x\right)\exp\left[-\frac{2\pi i3^{-p}\left(m^{\prime }p+n^{\prime }q\right)}{N}\right]$$

(14) $$\tilde{\Phi }\left(p^{\prime }\Delta x,q^{\prime }\Delta x\right)=\frac{a_{R}\sqrt{\Phi_{\phi }\left(p\Delta x,q\Delta x\right)}}{\Delta \kappa }$$

The total phase of the phase screen after sub-harmonic compensation is obtained by adding Equations (12) and (13). The phase screen generated by this method contains more low-frequency information, which can improve the large-scale statistical characteristics of the phase screen simulation.

### 2.2. Zernike Polynomial Method

Zernike polynomial is composed of an infinite sum of items, and each item is obtained by multiplying the coefficient and formula of the preceding item. The product plus sum is the final result. The polynomials form a complete orthonormal basis, each corresponding to a unique phase distortion, such as defocus, spherical aberration, etc. This algorithm is widely used in the surface simulation of optical components, and then gradually used in the simulation of the optical phase.

The polynomial is divided into two parts: the leading coefficient and the formula. The formula is obtained by multiplying the radial function $R_{n}^{m}\left(r\right)$ and the Angle function $\Theta_{n}^{m}\left(\theta \right)$. The coefficients are obtained indirectly by the covariance matrix.

The polynomial formula is as follows:

(15) $$S\left(r\right)=\sum_{j=1}^{\infty }a_{j}\cdot Z_{j}\left(r\right)$$

where $a_{j}$ is the leading coefficient, $Z_{j}\left(r\right)$ is written by [17]:

(16) $$Z_{even.j}=R_{n}^{m}\left(r\right)\sqrt{2\left(n+1\right)}\cos\left(m\theta \right),m\neq 0,\;Z_{odd.j}=R_{n}^{m}\left(r\right)\sqrt{2\left(n+1\right)}\sin\left(m\theta \right),m\neq 0,\;Z_{j}=R_{n}^{0}\left(r\right)\sqrt{n+1},m=0$$

where j is the serial number of the formula, n and m are radial series and angle series, respectively. Depending on the values of m and j, there are three forms. Radial function and angle function are as follows [18]:

(17) $$R_{n}^{m}\left(r\right)=\sum_{s=0}^{\frac{n-m}{2}}\frac{{\left(-1\right)}^{s}\left(n-s\right)!}{s!\left(\frac{n+m}{2}-s\right)!\left(\frac{n-m}{2}-s\right)!}r^{n-2s}$$

(18) $$\Theta_{n}^{m}\left(\theta \right)=\left\{\begin{array}{c} \sqrt{2\left(n+1\right)}\cos\left(m\theta \right),m\neq 0,j\;is\;even\\ \sqrt{2\left(n+1\right)}\sin\left(m\theta \right),m\neq 0,j\;is\;odd\\ \sqrt{n+1},m=0 \end{array}\right.$$

There is a certain correlation between the preceding coefficients, and they are not statistically independent. The covariance of $a_{j}$ and $a_{j}^{\prime }$ of any two coefficients of Zernike polynomial is [18]:

(19) $$\langle a_{j}a_{j}^{\prime }\rangle =\iint dfdf^{\prime }Q_{j}\left(f\right)\Phi_{\phi }\left(\frac{2f}{D},\frac{2f^{\prime }}{D}\right)Q_{j^{\prime }}\left(f^{\prime }\right)$$

(20) $$Q_{j}\left(f\right)=\iint d\rho d\theta Z_{j}\left(\rho ,\theta \right)e^{-i2\pi f\rho }$$

(21) $$\Phi_{\phi }\left(\frac{2f}{D},\frac{2f^{\prime }}{D}\right)={\left(4\pi \right)}^{2-\alpha }B\left(\alpha \right)c_{1}\left(\alpha \right){\left(\frac{D}{r_{0}}\right)}^{\alpha -2}f^{-\alpha }$$

where $r_{0}$ is the atmospheric coherence length and α is the power law of the three-dimensional spectrum, f is the spatial frequency of light wave, D is the diameter of the light beam.

(22) $$c_{1}\left(\alpha \right)=2{\left(\frac{8}{\alpha -2}\Gamma \left[\frac{2}{\alpha -2}\right]\right)}^{\frac{\alpha -2}{2}}$$

(23) $$B\left(\alpha \right)=\frac{\Gamma \left[\frac{\alpha }{2}\right]}{2^{2-\alpha }\pi \alpha \Gamma \left[-\frac{\alpha }{2}\right]}$$

The coefficient covariance matrix of Zernike polynomial is obtained by integral transformation:

(24) $$\langle a_{j}a_{j}^{\prime }\rangle =B\left(\alpha \right)c_{1}\left(\alpha \right)2^{5-2\alpha }\pi^{1-\alpha }{\left(\frac{D}{r_{0}}\right)}^{\alpha -2}\cdot {\left[\left(n+1\right)\left(n^{\prime }+1\right)\right]}^{\frac{1}{2}}{\left(-1\right)}^{\frac{n+n^{\prime }-2m}{2}}\delta_{mm^{\prime }}I_{nn^{\prime }}$$

(25) $$I_{nn^{\prime }}=\int f^{-\left(1+\alpha \right)}J_{n+1}\left(2\pi f\right)J_{n^{\prime }+1}\left(2\pi f\right)df=\frac{\Gamma \left[\frac{n+n^{\prime }-\alpha +2}{2}\right]}{\Gamma \left[\frac{n-n^{\prime }+\alpha +2}{2}\right]\Gamma \left[\frac{n^{\prime }-n+\alpha +2}{2}\right]}\cdot \frac{\pi^{\alpha }\Gamma \left[\alpha +1\right]}{2\Gamma \left[\frac{n+n^{\prime }+\alpha +4}{2}\right]}$$

The matrix $C_{a}$ composed of coefficient covariance is a real symmetric positive definite matrix, so singular value decomposition can be performed on it to obtain two matrices [18]:

(26) $$C_{a}=U\cdot \Lambda \cdot U^{T}$$

where Λ is diagonal matrix; U is the identity matrix, the transpose is the same as the inverse, $U=U^{T}=U^{-1}$.

There is a matrix X, whose elements $\left\{x_{i},i=1,2,3,\ldots ,\infty \right\}$ are Gaussian random variables with mean values of 0 and a variance determined by diagonal matrix, which satisfies the following relationship [18]:

(27) Y=U·X

where Y is the Zernike coefficient vector, and its elements are $\left\{y_{j},j=1,2,3,\ldots ,\infty \right\}$. Lastly, the Zernike polynomial can be expressed as:

(28) $$S\left(r\right)=\sum_{j=1}^{\infty }\sum_{i=1}^{\infty }x_{i}U_{ij}z_{j}\left(r\right)$$

### 2.3. Verification of Phase Screen Simulation

The phase structure function describes the second-order statistical characteristics of the wavefront phase and is often used as a method to check the phase screen. Both results of non-Kolmogorov turbulent phase screens generated by the power spectrum method and Zemike polynomial method are verified. Phase structure function is defined as [11]:

(29) $$D_{\phi }\left(r\right)={\langle \left[\phi \left(r+r_{1}\right)-\phi \left(r\right)\right]}^{2}\rangle$$

Non-kolmogorov turbulence phase structure function is expressed as [15]:

(30) $$D_{\phi }\left(r\right)=c_{1}\left(\alpha \right){\left(\frac{r}{r_{0}}\right)}^{\alpha -2}$$

where $c_{1}\left(\alpha \right)$ is expressed in the same way as Equation (22) above; $r_{0}$ means atmospheric coherence length, expressed as $r_{0}={\left(\frac{c_{1}\left(\alpha \right)\Gamma \left(\frac{\alpha }{2}\right)}{-2^{4-\alpha }\pi^{2}k^{2}A\left(\alpha \right)\Gamma \left(\frac{2-\alpha }{2}\right)\tilde{C_{n}^{2}}L}\right)}^{1/\left(\alpha -2\right)}$, when α=11/3, $c_{1}\left(11/3\right)=6.88$, $r_{0}={\left(0.423k^{2}C_{n}^{2}L\right)}^{-5/3}$, the phase structure function of non-Kolmogorov turbulence can be transformed into the phase structure function of Kolmogorov turbulence:

(31) $$D_{\phi }\left(r\right)=6.88{\left(\frac{r}{r_{0}}\right)}^{\frac{5}{3}}$$

#### 2.3.1. Verification of Turbulent Phase Screen Simulated by the Power Spectrum Method

**Figure 3:**
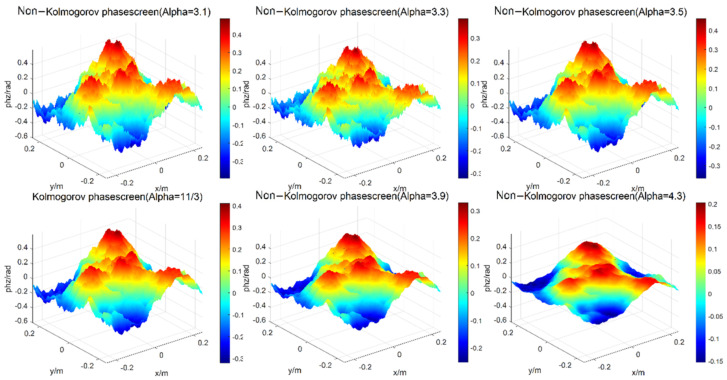
Simulations of Non-Kolmogorov turbulent phase screen by power spectrum method.

Firstly, a square phase screen with length and width of 0.5 m is simulated. When the number of grids is 256 × 256, the wavelength of light wave is 0.532 μm, the structure constant of atmospheric refractive index is $C_{n}^{2}=1.0\times 10^{-15}$ [19], the transmission distance is set as 1000 m, the phase screen spacing as ∆z = 100 m, regardless of the internal and external scales of turbulence, and the power laws of three-dimensional spectrum are taken as α = 3.1, 3.3, 3.5, 11/3, 3.9 and 4.3, respectively, the simulations of Non-Kolmogorov turbulent phase screen by the power spectrum method are shown in Figure 3.

As can be seen from the Figure 3, when the internal and external scales of turbulence are not considered and the same random number matrix is used, the phase disturbance generated by the phase screen of non-Kolmogorov turbulence is greatly affected by the power spectrum power law. It is not difficult to see that with the increase of power spectrum power law α, the phase fluctuation degree of non-Kolmogorov turbulence becomes smaller and smaller. Since the power spectrum law is a constant variable in the real atmosphere, it is often necessary to study the simulation results of laser beams under different power law conditions.

**Figure 4:**
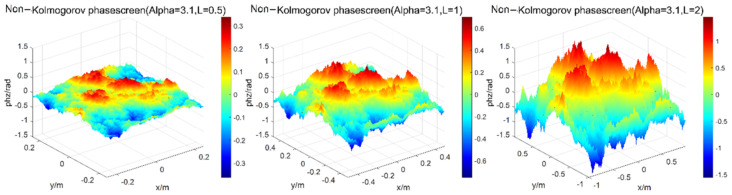
Simulations of phase screen by power spectrum method at different width of phase screen.

When changing the width of phase screen, namely, the grid number is 256 × 256, the wavelength of light wave is 0.532 μm, the atmospheric refractive index structure constant is $C_{n}^{2}=1.0\times 10^{-15}$, the transmission distance is set as 1000 and the phase screen spacing is set as ∆z = 100 m. Here, the power spectrum power law α = 3.1 is taken as an example. The width of phase screen is 0.5 m, 1.0 m, and 2.0 m, respectively, the simulation results of power spectrum are shown in Figure 4. 

**Figure 5:**
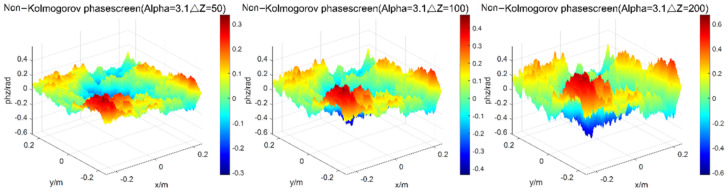
Simulations of phase screen by power spectrum method at different phase screen spacing.

When changing the phase screen interval, that is the width of phase screen is 0.5 m, the number of grids is 256 × 256, the wavelength of light wave is 0.532 μm, the atmospheric refractive index structure constant is $C_{n}^{2}=1.0\times 10^{-15}$, the transmission distance is set as 1000 m, the power spectrum power law α = 3.1, and the phase screen spacing is 50 m, 100 m, and 200 m, respectively, the simulation results of power spectrum are shown in Figure 5.

**Figure 6:**
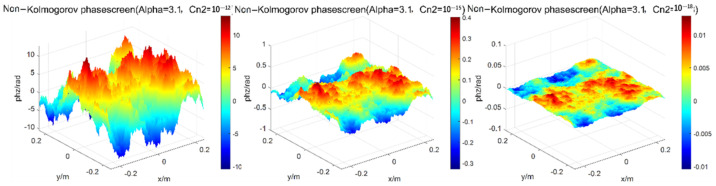
Simulations of power spectrum method under different turbulence intensity.

Change the intensity of atmospheric turbulence, the width of phase screen is 0.5 m, the number of grids is 256 × 256, the wavelength of light wave is 0.532 μm, the transmission distance is set as 1000 m, the distance of phase screen is set as 100 m, the power spectrum power law α = 3.1, the structure constants of atmospheric refractive index are $C_{n}^{2}=1.0\times 10^{-12}$, $1.0\times 10^{-15}$, and $1.0\times 10^{-18}$ [19], respectively, which representing three situations in which turbulence intensity is strong, medium, and weak, respectively. The simulation results of the power spectrum are shown in Figure 6.

As can be seen from the above simulation results, different phase screen widths, phase screen spacing and turbulence intensity have great changes in the spectral width of the phase screen and have great influences on the modeling and simulation of non-Kolmogorov turbulent phase screen. When the intensity of atmospheric turbulence is stronger, the width of the phase screen is larger, the distance between the phase screen is larger, and the phase fluctuation of the phase screen is larger. In practical applications, relevant parameters should be selected according to the laser emission system parameters and the actual situation of the atmospheric environment.

**Figure 7:**
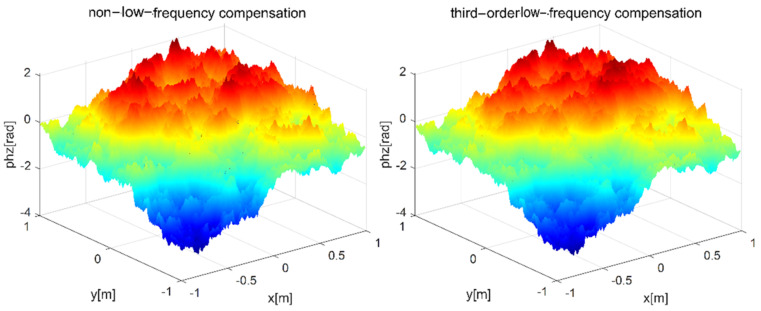
Simulations of power spectrum method under different harmonic waves.

**Figure 8:**
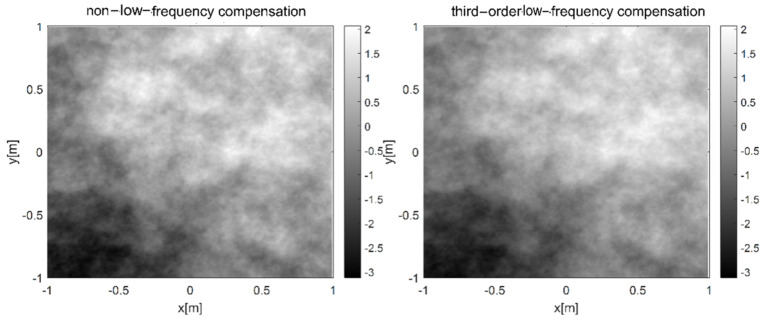
Grayscale of phase screen by power spectrum method at different harmonics.

The low frequency compensation is analyzed. When the wavelength of the light wave is set as λ = 0.532 μm, the phase screen spacing is set as 100 m, the transmission distance is set as 1000 m, the width of phase screen is 2 m, the number of grids is 256 × 256, the power spectrum power law α = 3.1, and the atmospheric refractive index structure constant is $C_{n}^{2}=1.0\times 10^{-15}$, the simulation results of non-low-frequency compensation and third-order low-frequency compensation are shown in Figure 7 and Figure 8, respectively.

As can be seen from the above two figures, the low-frequency part of the phase screen is not obvious when the third-order harmonic compensation is not added, resulting in inadequate sampling and affecting the authenticity of simulating non-Kolmogorov turbulence. After the third-order harmonic is added, the low-frequency components are compensated accordingly. The above two grayscale images are calculated, respectively. The average grayscale is 163.4397 and the variance is 5685.1 without harmonic compensation. After adding the third harmonic, the average gray level is 163.4473 and the variance is 5462.3. This shows that the image is smoother, and the variance relative error of the third harmonic is less than 5%.

Table 1 lists the influence of the subharmonic series is divided into several groups and statistically averaged, respectively.

It can be seen from Table 1 that the higher the subharmonic series is, the smaller the relative error of gray variance is. In theory, if the harmonic series continues to increase, the variance relative error will tend to 0, but in practice, it is usually set below 10 orders to meet the needs.

**Figure 9:**
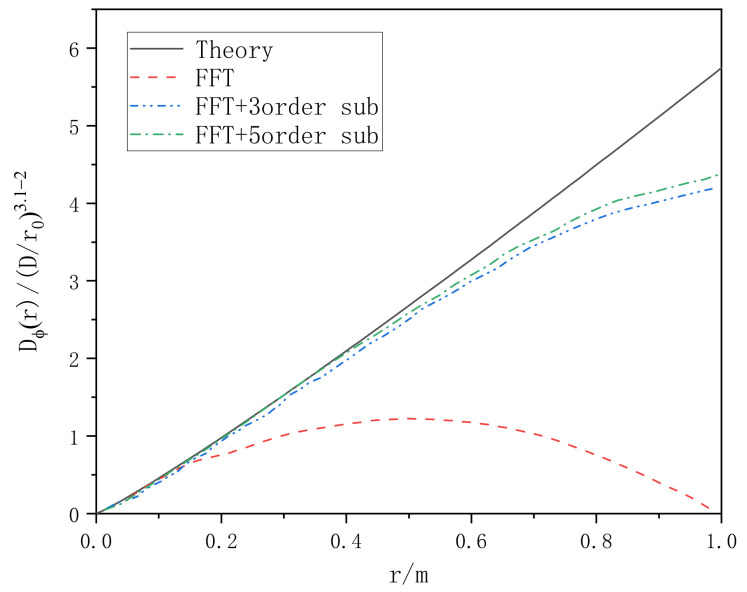
Phase structure function of power spectrum method for subharmonic compensation.

The structural function method is used to verify the effect of low frequency compensation. Taking power law α = 3.1 as an example, the phase structure function of a superimposed harmonic and non-superimposed harmonic is compared with the theoretical value, and the contrast function image is shown in Figure 9.

It can be seen from Figure 9 that the phase screen generated by the power spectrum method is close to the theoretical value in the high frequency part, and quite different from the theoretical value in the low frequency part. However, the phase screen generated by sub-harmonic compensation is significantly improved in the low spatial frequency part.

#### 2.3.2. Verification of Turbulent Phase Screen Simulated by Zernike Polynomial Method

**Figure 10:**
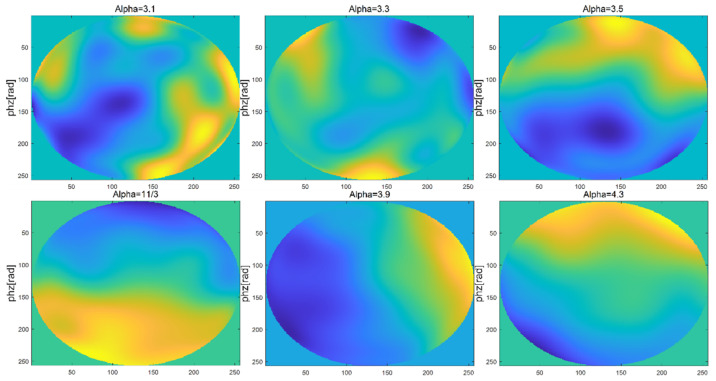
Simulations of non-Kolmogorov turbulent phase screen by the Zernike polynomial method.

When being similar to the simulation process of the power spectrum method, namely, the atmospheric coherence length $r_{0}$ = 0.1 m, phase screen width 0.5 m, grid number 256 × 256, light wave wavelength 0.532 μm, regardless of the internal and external scale of turbulence, the power law of the three-dimensional spectrum α = 3.1, 3.3, 3.5, 11/3, 3.9 and 4.3, respectively, and the order of Zernike polynomial was selected to be 10 for simulation, the simulations of non-Kolmogorov turbulent phase screen by the Zernike polynomial method are shown in Figure 10.

As can be seen from the figure, when the internal and external scales of turbulence are not considered and the Zernike polynomial order is the same, the phase disturbance generated by the phase screen of non-Kolmogorov turbulence is greatly affected by the power law. It can be seen that with the increase of power law α, the phase fluctuation degree of non-Kolmogorov turbulence becomes smoother and smoother.

**Figure 11:**
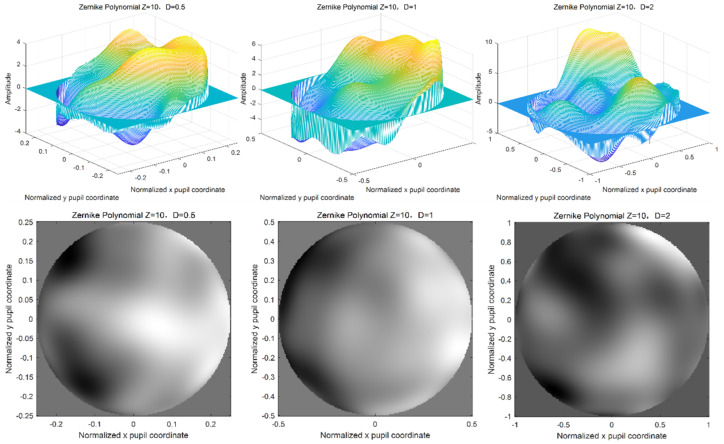
Simulation diagram of Zernike polynomial phase screen with different width of phase screen.

When changing the width of the phase screen, that is the number of grids is 256 × 256, the wavelength of light wave is 0.532 μm, and the atmospheric coherence length $r_{0}$ = 0.1 m. Here, the power law α = 3.1 is also taken as an example. The width of phase screen is 0.5 m, 1.0 m, and 2.0 m, respectively. Additionally, the polynomial order is 10. The simulations of Zernike polynomial phase screen with different widths of the phase screen are shown in Figure 11.

**Figure 12:**
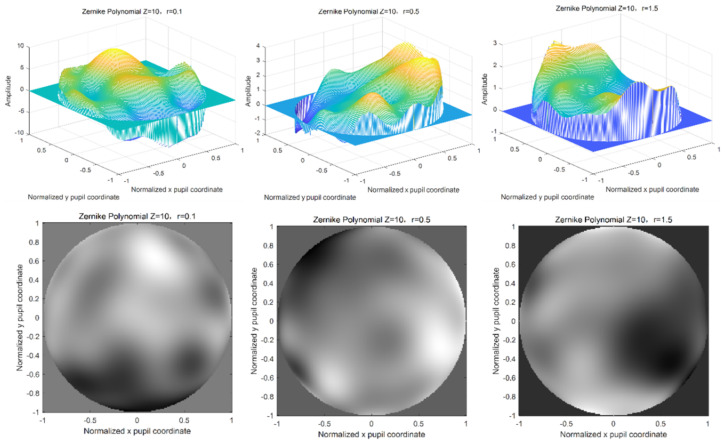
Simulation of Zernike polynomial phase screen with different atmospheric coherence lengths.

If changing the atmospheric coherence length, namely, the phase screen width is 0.5 m, the mesh number is 256 × 256, the light wave wavelength is 0.532 μm, the power law α = 3.1, the polynomial order is 10, and the atmospheric coherence length is $r_{0}$ = 0.1 m, 0.5 m, and 1.5 m, respectively, the simulations of Zernike polynomial phase screen with different atmospheric coherence lengths are shown in Figure 12.

It can be seen from the above simulation results that different phase screen widths and different atmospheric coherence lengths have great changes in the amplitude width of the phase screen and have great influences on the modeling and simulation of the non-Kolmogorov turbulent phase screen. When the atmospheric coherence length is smaller and the phase screen width is larger, the phase fluctuation of the phase screen will be larger. In practical application, relevant parameters should be selected according to the laser emission system parameters and the actual situation of the atmospheric environment.

**Figure 13:**
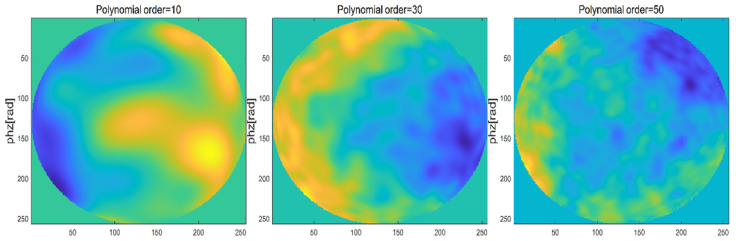
Simulation of Zernike polynomial phase screen with different orders.

When changing the order of Zernike polynomial, that is the phase screen width is 0.5 m, the mesh number is 256 × 256, the wavelength of light wave is 0.532 μm, the atmospheric coherence length $r_{0}$ = 0.1 m, the power law α = 3.1, and the orders are 10, 30 and 50, respectively, the simulations of Zernike polynomial phase screen with different orders are shown in Figure 13.

**Figure 14:**
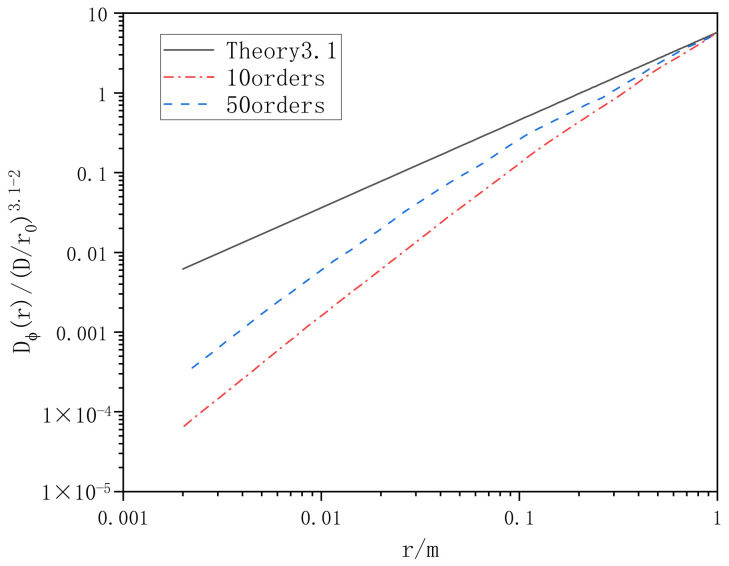
Zernike polynomial phase structure function.

In order to analyze the performance of the phase screen generated by Zernike polynomial method, the phase structure function was also used for statistical analysis. The phase screen generated by several different polynomial orders was statistically analyzed and compared with the theoretical value of atmospheric turbulence phase structure function. The comparison results are shown in Figure 14.

It can be seen from Figure 13 and Figure 14 that the Zernike polynomial method is insufficient to simulate the high frequency information of non-Kolmogorov turbulence. When the order of Zernike polynomial is increased, the phase structure function is closer to the theoretical value, and the high frequency information is slightly improved.

Both the power spectrum method of sub-harmonic compensation and Zernike polynomial method of increasing order can increase the accuracy of phase screen simulation to a certain extent. In the actual simulation operation, we compared the running time of the two methods. The running time of the power spectrum method was about 3 s, while the running time of the Zernike polynomial method was more than 10 s, and the higher the order, the longer the running time. After comprehensive consideration, the power spectrum method with sub-harmonic compensation was selected for subsequent simulation.

## 3. Simulation of Atmospheric Turbulence Detection by Mie-Scattering Lidar Using Non-Kolmogorov Turbulence Power Spectrum

The main objects of theoretical analysis and processing of turbulent atmospheric optics are ideal plane waves and spherical waves, while the tool or main research object of turbulent atmospheric optics experiment is the wave of a laser beam. The difference between laser and general light waves is not only reflected in the laser’s unique coherence and high brightness, but also in that the laser is a beam of limited space, so there is a clear spot. 

For the Mie-scattering lidar system detecting atmospheric turbulence, Gaussian beam is emitted by laser, and the atmospheric turbulence profile is obtained by using return signals at different heights. However, the transmission characteristics of a laser in the atmosphere will affect the actual detection performance of the system. Therefore, the influence of atmosphere on laser transmission characteristics must be considered in the design and application of the Mie-scattering lidar system.

The most direct effect of turbulent atmosphere on laser propagation is the spatial distribution of light intensity, that is, the change of spot shape. To study the difference between the simulation using non-Kolmogorov turbulence and Kolmogorov turbulence for Mie-scattering lidar, this paper conducts simulation tests on the detection technology based on the detection principle of Mie-scattering lidar. 

### 3.1. Mie-Scattering Lidar System

**Figure 15:**
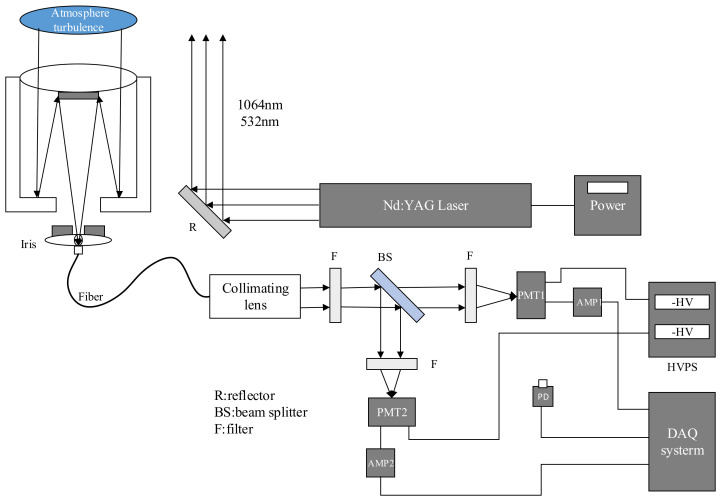
Schematic diagram of the Mie-scattering lidar system.

Figure 15 shows the schematic diagram of the Mie-scattering lidar system used. The system uses the Nd: YAG-pulsed laser as the laser source, and emits the pulsed laser beam at wavelengths of 1064 nm and 532 nm into the atmosphere, then the laser interacts with the molecules and particles in the atmosphere as well as the atmospheric turbulence to generate the backscattering return signal, which is received by the large aperture telescope. Through the iris, optical fiber and collimating lens, the return signal incidents into the spectroscopic system, in which, the 532 nm return signal is divided into two channels with different energies and is, respectively, detected by two photomultiplier tubes (PMT). The purpose of the two channels is to explore the effects of return signals with different intensities on atmospheric turbulence in later experiments. The PMTs convert the received optical signals into electrical signals, which are amplified by amplifiers and sent to the data acquisition and processing system. The photoelectric detector of the lidar works in the analog mode, and the output of PMT is directly connected with the high speed, high gain and low noise amplifier circuit to achieve impedance matching and signal amplification.

The beam emitted by the Mie-scattering lidar is a Gaussian beam, and the amplitude and intensity of the light field of the Gaussian beam are Gaussian distributed in a plane perpendicular to the direction of propagation. The field of the fundamental mode Gaussian beam in a uniform medium can be expressed as [1]:

(32) $$U\left(x,y,z\right)=U_{0}\frac{\omega_{0}}{\omega \left(z\right)}exp\left\{-\left[\frac{r^{2}}{\omega^{2}\left(z\right)}+i\frac{kr^{2}}{2R\left(z\right)}\right]\right\}$$

(33) $$I\left(x,y,z\right)=U_{0}^{2}\frac{\omega_{0}^{2}}{\omega^{2}\left(z\right)}exp\left\{-\frac{2r^{2}}{\omega^{2}\left(z\right)}\right\}$$

where $\omega \left(z\right)=\omega_{0}\sqrt{1+{\left(\frac{z}{f}\right)}^{2}},f=\frac{\pi \omega_{0}^{2}}{\lambda },\omega_{0}=\sqrt{\frac{\lambda f}{\pi }},R=R\left(z\right)=z\left[1+{\left(\frac{z}{f}\right)}^{2}\right]=z+\frac{f^{2}}{z}$, $\omega_{0}$ represents the optical waist radius, $R\left(z\right)$ is the radius of wave-front curvature, $U_{0}$ represents the initial light field distribution of the fundamental mode Gaussian beam, and f represents the confocal parameter.

### 3.2. Light Intensity Simulation of Gaussian Beam Propagation for Non-Kolmogorov Turbulence

According to Equations (32) and (33), the change of intensity of Gaussian beam propagating 1 km in turbulent atmosphere is simulated. Therefore, the laser is set as fundamental mode Gaussian beam, taking laser wavelength λ = 0.532 μm, phase screen size L = 0.5 m, beam waist radius $\omega_{0}$ = 20 mm, grid number N × N = 256 × 256, and setting a phase screen at Δz = 100 m in the distance of 1000 m, the power law α = 3.1, 3.5, and 3.9, the atmospheric refractive index structure constants are $C_{n}^{2}=1.0\times 10^{-12}$ and $C_{n}^{2}=1.0\times 10^{-15}$, respectively. The power spectrum method is employed to simulate the distribution of light intensity. In fact, the laser used in the laboratory has wavelengths of 1064 nm and 532 nm, energy of 150 mJ, frequency of 10 Hz, and average power of 1.5 W. The initial light intensity is the ratio of the power to the phase screen area, which is 6 W/m^2^ through calculation. It should be pointed out that the light intensity calculated by simulation in the following figure is a relative value, which is the ratio of the light intensity of each point to the origin.

**Figure 16:**
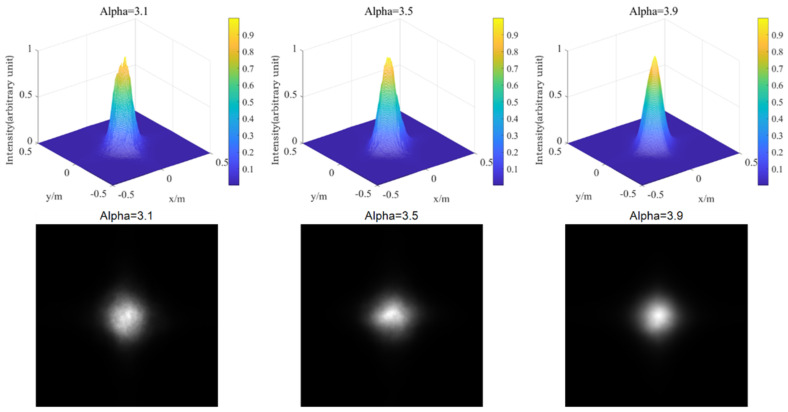
Light intensity distribution of different power law values when $C_{n}^{2}=1.0\times 10^{-15}$.

Firstly, the phase screen of laser transmission under medium intensity turbulence is simulated ($C_{n}^{2}=1.0\times 10^{-15}$), as shown in Figure 16.

**Figure 17:**
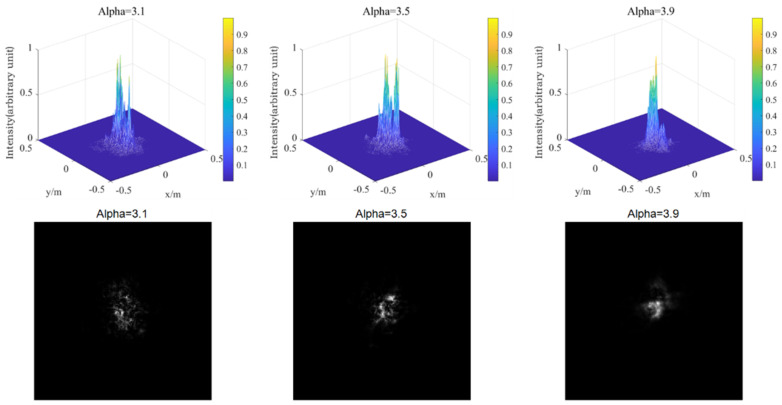
Light intensity distribution of different power law values when $C_{n}^{2}=1.0\times 10^{-12}$.

Then, the phase screen of laser transmission under strong turbulence is simulated ($C_{n}^{2}=1.0\times 10^{-12})$, as shown in Figure 17.

As can be seen from Figure 16 and Figure 17, for non-Kolmogorov turbulence, the smaller power law of the three-dimensional power spectrum is, the larger the degree of spot breakage of the Gaussian beam is. This shows that the power law has a great influence on laser transmission. In practical application, the selection of appropriate power spectrum power law value has a great effect on the simulation results. In the transmission process of Gaussian beam in turbulent atmosphere, the light spot of Gaussian beam gradually breaks with the increase of the atmospheric structure constant. With the increase of turbulence intensity, the degree of light spot breakage becomes more obvious, and the radius of beam also increases. This indicates that the stronger the turbulence intensity is, the greater the distortion is to the laser transmission, which not only affects the overall intensity of the light intensity, but also affects the uniformity of the light intensity. In practice, atmospheric turbulence is usually composed of turbulence with a different power law, which will have a great impact on the effect of laser transmission, and then affect the near-field beam quality. In some satellite communication, lasers ranging and other aspects, the impact of turbulence cannot be ignored, and should be analyzed according to the actual situation.

Light intensity fluctuation is one of the most common and obvious light transmission effects caused by atmospheric turbulence [20]. The random fluctuation of laser intensity with time is called intensity fluctuation when laser propagates in a turbulent atmosphere. The fluctuation is caused by the random fluctuation of atmospheric refractive index which leads to the change of laser phase and the random fluctuation of laser amplitude. Light intensity scintillation is an important physical quantity that restricts the quality of laser communication. The scintillation index $\sigma_{I}^{2}$ represents the normalized light intensity fluctuation variance, which is defined as [21]:

(34) $$\sigma_{I}^{2}=\frac{\langle I^{2}\rangle -\langle I\rangle^{2}}{\langle I\rangle^{2}}$$

where I represents light intensity, and 〈·〉 represents ensemble average.

The weak fluctuation condition of atmospheric turbulence can be obtained by using the Rytov approximate relation ($\sigma_{lnI}^{2}\approx \sigma_{Rytov}^{2}$), $\sigma_{I}^{2}<1$. The normalized light intensity fluctuation variance corresponding to the plane wave under the weak fluctuation condition is usually used as the measurement parameter of the fluctuation condition, which is called the Rytov index [1]:

(35) $$\sigma_{Rytov}^{2}\left(L\right)=1.23k^{\frac{7}{6}}L^{\frac{11}{6}}C_{n}^{2}$$

When laser is transmitted in weakly fluctuating turbulence, the scintillation index is $\sigma_{I}^{2}$, the logarithmic amplitude fluctuation variance is $\sigma_{lnI}^{2}$ and the Rytov fluctuation variance $\sigma_{Rytov}^{2}$ satisfy the following requirements:

(36) $$\sigma_{I}^{2}=\sigma_{lnI}^{2}=\exp\left(\sigma_{Rytov}^{2}\right)-1$$

It can be seen that when $\sigma_{I}^{2}$ increases gradually, $\sigma_{Rytov}^{2}$ also increases gradually. When the scintillation index $\sigma_{I}^{2}$ increases to more than one, the saturation phenomenon of light intensity scintillation will appear. In this case, although $\sigma_{Rytov}^{2}$ increases, the scintillation index $\sigma_{I}^{2}$ starts to decrease. Markov approximation is introduced to solve the approximate solution of scintillation mean square error under strong fluctuation condition $\sigma_{I}^{2}\geq 1$.

For Gaussian beam, its scintillation index is expressed as [22]:

(37) $$\sigma_{I}^{2}\left(L\right)=\sigma_{lnI}^{2}\left(L\right)=\;8\pi^{2}k^{2}L\int_{0}^{1}\int_{0}^{\infty }\kappa \phi_{n}\left(\kappa \right)\exp\left(-\frac{\Lambda Lk^{2}\xi^{2}}{k}\right)\{1-\cos[\frac{L\kappa^{2}}{k}\xi \left(1-\bar{\Theta }\xi \right]\}d\kappa d\xi$$

where ξ=z/L, Θ=1+L/F, $\bar{\Theta }=1-\Theta$, $\Lambda =2L/k\omega^{2}$, F represents the wave-front curvature radius of the receiving plane, ω represents the beam diameter at the receiving plane.

**Figure 18:**
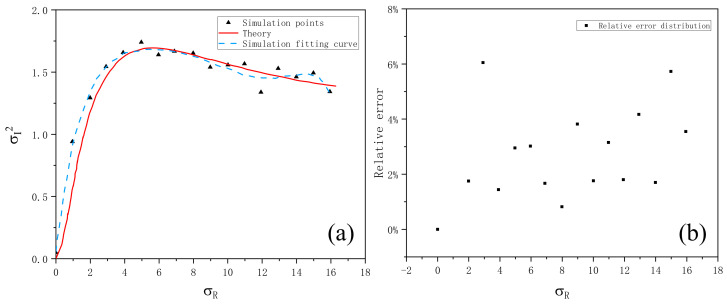
(**a**) The variation of scintillation index with Rytov variance simulated by power spectrum method. (**b**) Relative error of scintillation index with Rytov variance.

Then, the phase screen method is used to simulate the scintillation index of laser transmission using non-Kolmogorov turbulent media. When the Gaussian beam wavelength is λ = 0.532 μm, the phase screen size is 0.5 m, the beam waist radius is $\omega_{0}$ = 20 mm, the number of grids is N × N = 256 × 256, the transmission height is L = 500 m, and phase screen interval is Δz = 200 m, the scintillation index as a function of Rytov variance is simulated under the condition of non-Kolmogorov turbulent atmosphere, as shown in Figure 18.

From Figure 18a, the simulated scintillation index in non-Kolmogorov turbulent atmosphere is in good agreement with the theoretical value. With the continuous increase of Rytov variance, the scintillation index first increases to a maximum value, then slightly decreases, and finally tends to a stable value, which is the saturation phenomenon of light intensity scintillation mentioned above. Figure 18b shows the relative error between simulated non-Kolmogorov turbulent atmospheric scintillation index and theoretical value. It can be seen that the error is basically less than 10%, and the average relative error is 2.66% after calculation, which verifies the accuracy of simulation.

**Figure 19:**
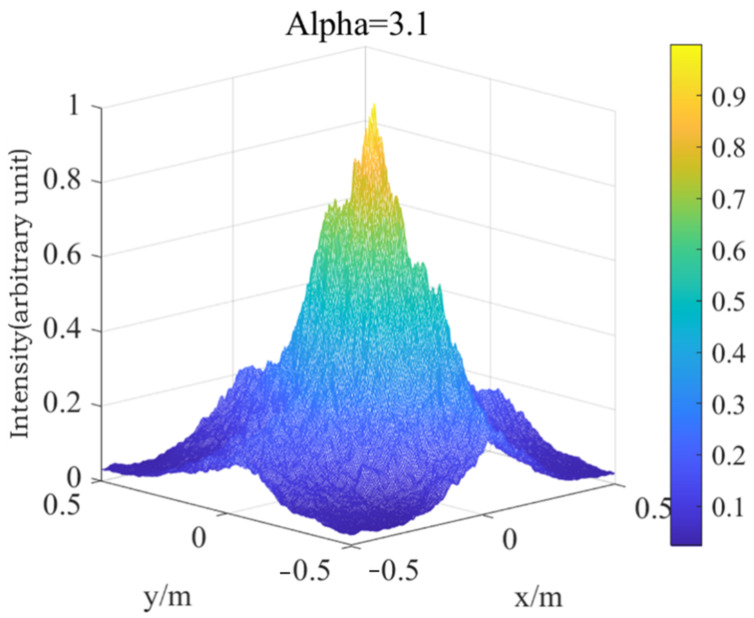
Light intensity distribution of Gaussian beam propagating vertically at 5000 m in non-Kolmogorov turbulence.

**Figure 20:**
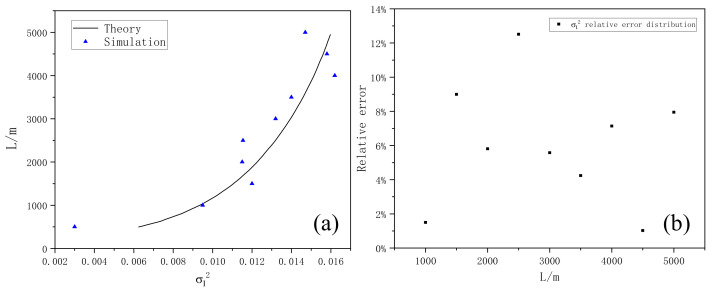
(**a**) The comparison between the average value of the scintillation index and the theoretical value of the Gaussian beam transmitted by the Mie scattering lidar at the vertical height of 5000 m. (**b**) Relative error of scintillation index with height.

Figure 19 and Figure 20 show the three-dimensional light intensity distribution and the comparison between the average value of the scintillation index and the theoretical value of the Gaussian beam transmitted by the Mie scattering lidar at the vertical height of 5000 m, using the power spectrum method.

As can be seen in Figure 19 and Figure 20, the simulated scintillation index fluctuates around the theoretical value, and there is a good consistency between them, which preliminarily indicates the reliability and rationality of Mie scattering lidar when using non-Kolmogorov turbulence and can play a good guiding role in the subsequent practical detection. Figure 20b shows the relative error of simulated scintillation index with height. It can be seen that the relative error is basically less than 14% and most of it is less than 8%, indicating that it is feasible to use the parameters of the Mie-scattering lidar to simulate the fluctuation of light intensity. In the future, we will use simulated parameters for the equipment design and construction of the lidar system.

### 3.3. Spot Drift Effect of Gaussian Beam Propagation in Non-Kolmogorov Turbulence

When the laser is transmitted in the turbulent atmosphere, the center position of the statistical average of the beam will have a fast random jump in the plane perpendicular to its transmission direction. This phenomenon is called spot drift. This phenomenon is the most common deformation characteristic of light beams in a turbulent atmosphere. Spot drift effect has important influence on laser communication, laser ranging and lidar systems.

Spot drift is usually described by the change of spot centroid position. There are two methods to detect the centroid of the spot. One is the centroid method which starts from the gray level distribution inside the spot, which needs to calculate every pixel on the spot. The other is edge detection method starting from spot edge information, such as Hough transform and least square method fitting spot contour, etc. [23]. Due to the fact that light spot image is obtained by simulation of phase screen will be broken and discrete degree is bigger with the increase of transmission distance and phase screen spacing, the edge information is difficult to obtain. In fact, the information of light field intensity change can be clearly obtained by using the simulation model of Gaussian beam and further can be linearly transformed into pixel points on the phase screen. Therefore, the centroid method is used to solve the centroid distribution of light spots. The centroid of the spot is defined as [1]:

(38) $$\left\{\begin{array}{c} x_{c}=\frac{\iint xI\left(x,y\right)dxdy}{\iint I\left(x,y\right)dxdy}\\ y_{c}=\frac{\iint yI\left(x,y\right)dxdy}{\iint I\left(x,y\right)dxdy} \end{array}\right.$$

So, the drift variance of the center of mass is:

(39) $$\sigma_{\rho }^{2}=\langle \rho_{c}^{2}\rangle =\frac{\iint \iint \left(\rho_{1}\cdot \rho_{2}\right)I\left(\rho_{1}\right)I\left(\rho_{2}\right)d\rho_{1}d\rho_{2}}{{\left[\iint I\left(\rho \right)d\rho \right]}^{2}}$$

If the mean square deviation of the spot centroid drift in the horizontal and vertical directions is $\sigma_{x}$ and $\sigma_{y}$, respectively, and under the assumption that the drift motion in the horizontal and vertical directions is statistically independent, the total drift variance of the spot centroid is given by:

(40) $$\sigma_{\rho }^{2}=\sigma_{x}^{2}+\sigma_{y}^{2}$$

When the Gaussian beam wavelength λ = 0.532 μm, the phase screen size L = 0.5 m, the beam waist radius $\omega_{0}$ = 20 mm, and the mesh number N × N = 256 × 256 are selected, the transmission distance is 1 km, a phase screen interval is set as Δz = 100 m, and the power law is set as α = 3.1 and 3.9, respectively, and the atmospheric refractive index structure constants are $C_{n}^{2}=1.0\times 10^{-12}$, $C_{n}^{2}=1.0\times 10^{-15}$, the distribution of spot centroid is simulated.

**Figure 21:**
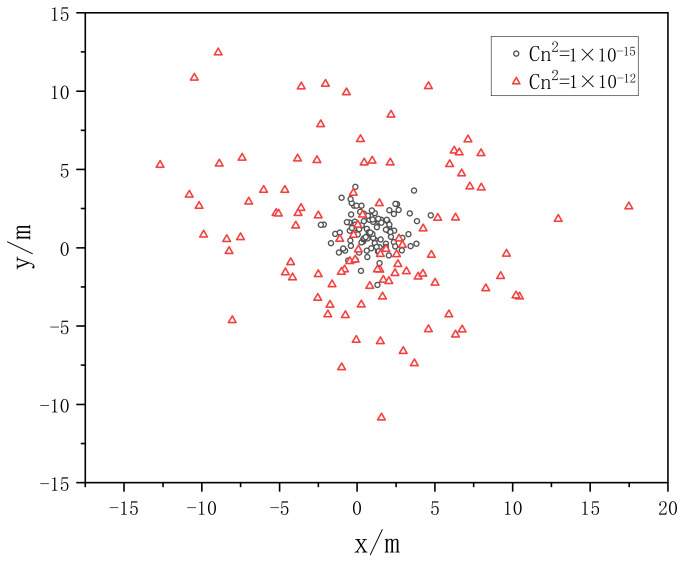
The distribution of 100 centroids randomly sampled when *α* = 3.1.

**Figure 22:**
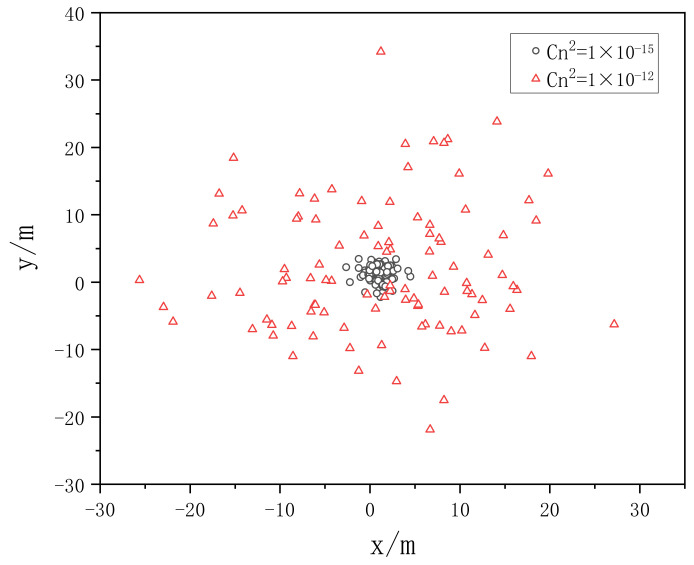
The distribution of 100 centroids randomly sampled when *α* = 3.9.

Figure 21 and Figure 22 show the distribution of 100 centroids randomly sampled after Gaussian beam propagates in different turbulence when α = 3.1 and 3.9, respectively. It can be seen from the two figures that with the increase of the turbulent atmospheric structure constant, the spot drift effect becomes more obvious. When the turbulent atmospheric structure constant remains unchanged, the larger the power spectral power law, the more obvious the spot drift effect becomes.

Therefore, it can be concluded that the comprehensive influence under each power law should be considered comprehensively in the subsequent practical lidar detection. The degree of fragmentation of light spot mainly comes from the influence of large power law spectrum, and it needs to be systematically analyzed in the experimental detection to ensure the accurate source of laser distortion.

## 4. Conclusions

In this paper, the feasibility of detecting atmosphere turbulence with Mie-scattering lidar is verified by numerical simulation. Based on the theory of non-Kolmogorov turbulence spectrum model, the non-Kolmogorov turbulence phase screen was constructed by using power spectrum method and Zernike polynomial method, respectively, and the relevant parameters were changed to verify the accuracy of the phase screen. By comparison, the power spectrum method was selected to simulate the subsequent laser propagation.

The feasibility of detecting atmospheric turbulence by Mie-scattering lidar is evaluated by using non-Kolmogorov atmospheric turbulence model by simulation. The simulation results show that the smaller the power law is, the stronger the turbulence intensity is, and the more the beam intensity is affected by turbulence. The larger the power law is, the larger the spot drift is. The scintillation index of simulated Gaussian beam propagating vertically for non-Kolmogorov turbulence is compared with the theoretical value. It shows the reliability and rationality of detecting atmospheric turbulence by using Mie-scattering lidar. The simulation results preliminatively prove the reliability of Mie-scattering lidar in detecting atmospheric turbulence, which can play a guiding role in subsequent practical detection. In future, the Mie-scattering lidar will be developed according to the parameters resulted from the simulation.

## Figures and Tables

**Table 1:** Influence of subharmonics on image gray scale.

Group Number	Subharmonics	Mean Gray Scale	Variance of Gray Scale	Compensated Variance Relative Error
1	0	163.4397	5681.5	3.92%
	3	163.4473	5462.3	
2	0	177.8483	5531.5	2.06%
	6	177.8651	5417.8	
3	0	159.8466	6116.3	1.42%
	10	159.8518	6029.4	
4	0	181.1989	4540.2	0.72%
	15	181.2009	4507.3	

## Data Availability

The relevant data used to support the findings of this study are available from the corresponding author upon request.
